# Transcapillary transport of water, small solutes and proteins during hemodialysis

**DOI:** 10.1038/s41598-020-75687-1

**Published:** 2020-10-30

**Authors:** Leszek Pstras, Jacek Waniewski, Bengt Lindholm

**Affiliations:** 1 Nalecz Institute of Biocybernetics and Biomedical Engineering, Polish Academy of Sciences, Ks. Trojdena 4, 02-109 Warsaw, Poland; 2 Division of Renal Medicine and Baxter Novum, Department of Clinical Science, Intervention and Technology, Karolinska Institute, Stockholm, Sweden

**Keywords:** Computational models, Haemodialysis, Biomedical engineering, Circulation

## Abstract

The semipermeable capillary walls not only enable the removal of excess body water and solutes during hemodialysis (HD) but also provide an essential mechanism for maintaining cardiovascular homeostasis. Here, we investigated transcapillary transport processes on the whole-body level using the three-pore model of the capillary endothelium with large, small and ultrasmall pores. The transcapillary transport and cardiovascular response to a 4-h hemodialysis (HD) with 2 L ultrafiltration were analyzed by simulations in a virtual patient using the three-pore model of the capillary wall integrated in the whole-body compartmental model of the cardiovascular system with baroreflex mechanisms. The three-pore model revealed substantial changes during HD in the magnitude and direction of transcapillary water flows through small and ultrasmall pores and associated changes in the transcapillary convective transport of proteins and small solutes. The fraction of total capillary hydraulic conductivity attributed to ultrasmall pores was found to play an important role in the transcapillary water transport during HD thus influencing the cardiovascular response to HD. The presented model provides a novel computational framework for a detailed analysis of microvascular exchange during HD and as such may contribute to a better understanding of dialysis-induced changes in blood volume and blood pressure.

## Introduction

Microvascular exchange of water and solutes between the blood and tissues plays a key role in the function and homeostasis of the human body. The transport properties of the exchange vessels vary between different organs and tissues depending on the type of capillaries (continuous, fenestrated or sinusoidal) and may change in some pathological conditions^1^. The magnitude and direction of transcapillary water and solute transport does also change with time depending on the physiological circumstances. On the whole-body level, under steady-state conditions, there is a net filtration of water and solutes (including proteins) from the blood to the tissues, which is compensated by the equivalent lymphatic absorption of interstitial fluid and its transport back to the circulation in the form of lymph^2,3^. During hemodialysis (HD), when relatively large quantities of water and solutes are removed from the patient’s body over a few hours, the normal transcapillary filtration becomes progressively reduced and eventually reverses into absorption of fluid from the interstitial space in order to compensate for the reduced blood volume to maintain cardiovascular stability (vascular refilling mechanism^4–7^). Given that intradialytic hypotension remains the major complication in HD^8,9^, study of transcapillary shifts of water and solutes, in particular proteins, may provide important insights to the understanding of this highly undesired phenomenon and could potentially contribute to improvements in the safety and effectiveness of HD therapy.

**Figure 1:**
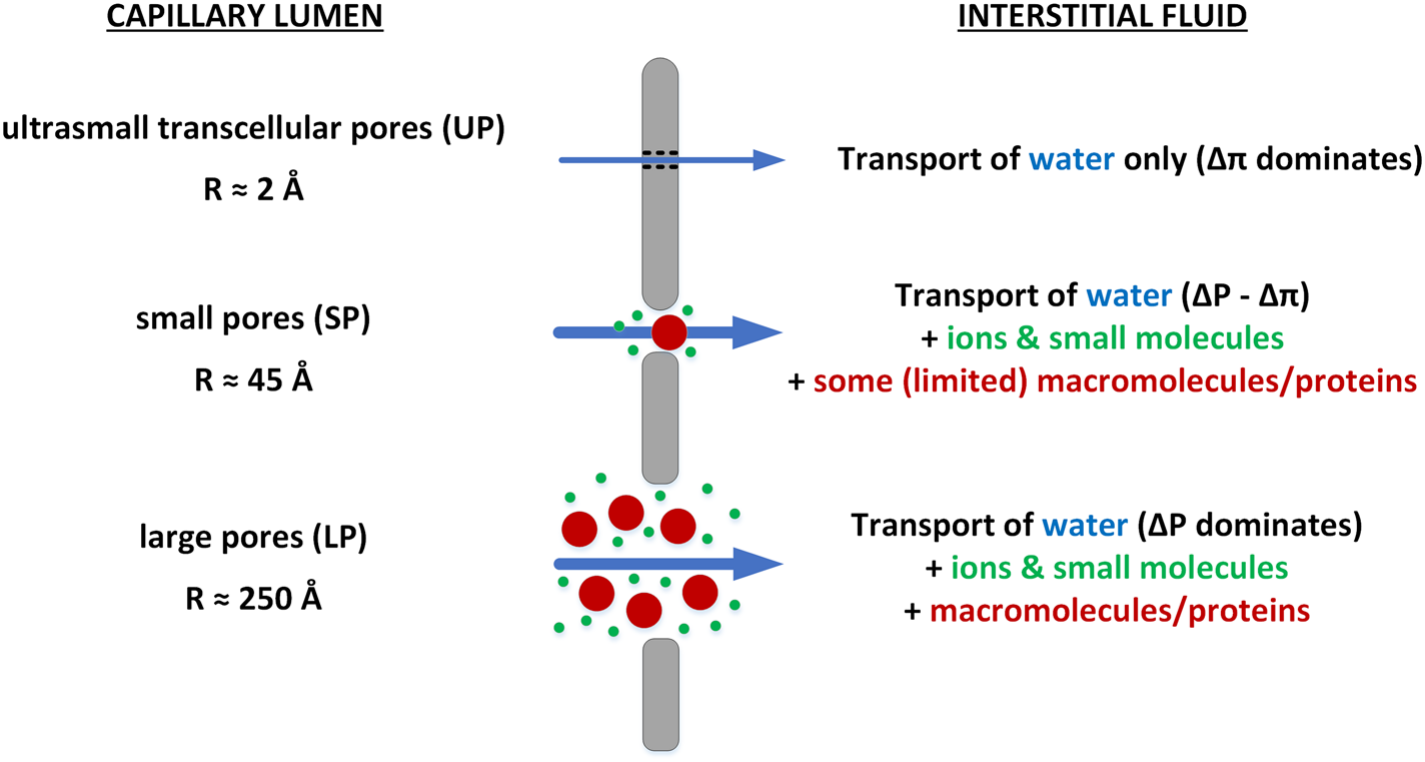
The three-pore model of the transcapillary transport of water, small solutes and macromolecules. The transport of water is driven by the hydraulic-hydrostatic pressure difference between capillary blood and interstitial fluid (∆P) counteracted by the osmotic pressure difference between plasma and interstitial fluid (∆π).

Over time, there have been multiple approaches to mathematically model the microvascular exchange processes^10,11^. For the continuous capillaries, which are dominant in the human body (e.g. in skeletal muscles, skin, fat tissue, heart and lungs), the currently accepted and widely used concept is the three-pore model (3PM)^12,13^, which attempts to reflect the structural properties of continuous capillaries by identifying three separate channels for solute and/or water transport (see Fig. 1): (a) large pores (LP) with radius ~ 250 Å representing large inter-endothelial gaps or junctions that allow an almost unobstructed passage of most plasma macromolecules; (b) small pores (SP) with radius ~ 45 Å representing clefts between the endothelial cells allowing the passage of all ions and small solutes and a limited passage of small proteins, such as albumin; and (c) ultrasmall pores (UP) with radius ~ 2 Å representing transcellular aquaporins that allow only water to pass^12,13^.

Given that different pores leak different amounts of proteins, depending on their reflection (or sieving) coefficients, the colloid osmotic pressure gradient develops to a different extent across different types of pores (the same, albeit much smaller effect applies to the osmotic pressure gradient exerted by the small solutes). Hence, despite the same hydraulic–hydrostatic pressure difference acting across all types of pores, the actual rate of fluid filtration through different pores may not reflect their relative hydraulic capacity^14^. For instance, a very low oncotic pressure difference develops across the highly-permeable large pores, and hence a relatively large filtration should be expected to occur via this transport channel, despite its relatively low area and low hydraulic conductivity. Under non-steady conditions, such as during HD, the magnitude and direction of water and solute flow through different pores may vary substantially.

The aim of this study was to investigate the microvascular fluid shifts on the whole-body level using the 3PM and to assess the impact of relative hydraulic capacities of individual types of capillary pores on the overall cardiovascular response to HD.

## Materials and methods

### Model overview

**Figure 2:**
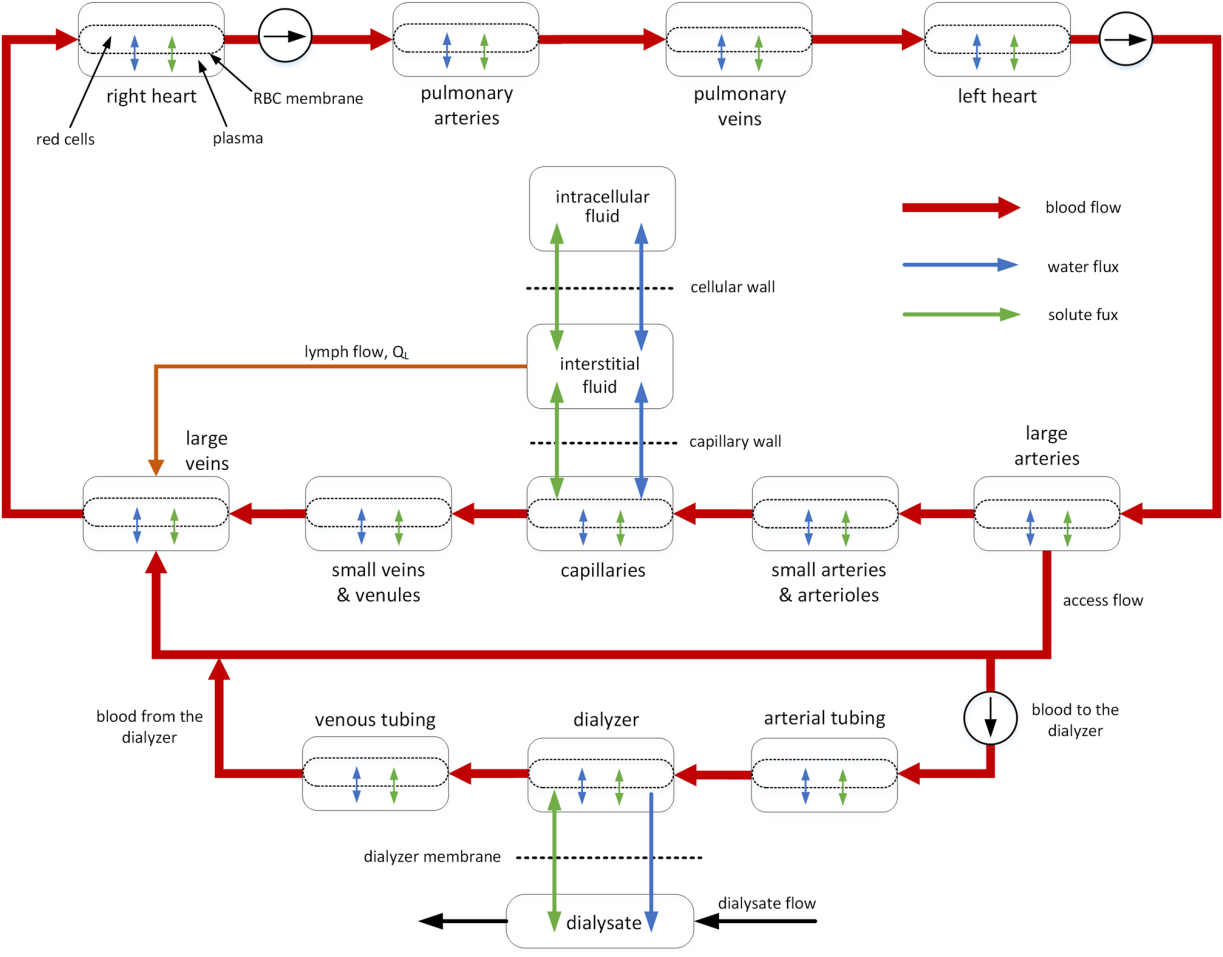
Overview of the integrated model of the cardiovascular system, extracorporeal dialyzer circuit and the whole-body water and solute exchange (based on Pstras and Waniewski^17^).

The simulations shown is this study are based on our earlier lumped-parameter compartmental model of the human cardiovascular system with baroregulation^15,16^ integrated with the model of the whole-body water and solute transport designed for modeling of the cardiovascular response to HD^17^. The proposed model is a non-pulsatile model describing the two-phase blood flow (plasma with suspended erythrocytes) across nine cardiovascular compartments and three extracorporeal compartments, as shown in Fig. 2. The cardiovascular model is equipped with four baroreflex mechanisms controlling peripheral resistance, heart rate, heart contractility and venous unstressed volume. The model includes the aggregated whole-body transport of water and solutes across the capillary wall, tissue cell membrane and erythrocyte membrane as well as via the lymphatic system. The solutes considered in the model include ions (Na^+^, K^+^, Cl^−^, HCO_3_^−^, and other cations and anions treated collectively), small solutes (urea and creatinine) and proteins (albumin and all other proteins treated collectively as globulins).

The model describes changes in pressures, volumes and composition of all considered fluids in individual compartments (blood, plasma, interstitial fluid, intracellular fluid). The model is based on the set of ordinary differential equations and was implemented in MATLAB (The MathWorks Inc.). For a full description of the model and all model parameters, see our earlier work^17^.

### Transcapillary transport model

In terms of transcapillary transport, our original model^17^ uses the classic homogeneous membrane model, which for the purpose of this study was replaced by the 3PM. The rate of fluid exchange between the capillary compartment and the interstitial compartment was calculated separately for each type of pores depending on the imbalance between the Starling forces acting across the pores: the hydraulic capillary blood pressure, the hydrostatic interstitial pressure and the osmotic (mainly oncotic) pressures exerted by all solutes on both sides of the capillary wall (see Eq. 1 in the Supplementary Information S1).

The radii of large, small and ultrasmall pores were assumed at 250 Å, 45 Å and 2 Å, respectively^18^. The total capillary hydraulic conductivity (LpS) was assumed at 4.5 mL/min/mmHg^4,19^, of which 5%, 85%, and 10% were attributed to LP, SP, and UP, respectively. Based on the assumed pore radii^18^ and hydrodynamic (Stokes–Einstein) solute radii, the reflection coefficients and permeability-surface products (PS) were calculated for all solutes for small and large pores, as shown in Table 1. For all equations describing the 3PM, see the Supplementary Information S1 available online.

**Table 1:** Assumed hydrodynamic (Stokes–Einstein) solute radii (r_s_)^21–28^ and calculated reflection coefficients (σ) and permeability-surface products (PS) for large pores (LP) and small pores (SP).

	r_s_ [Å]	σ_LP_	σ_SP_	PS_LP_ [mL/s]	PS_SP_ [mL/s]
Sodium (Na^+^)	1.8	0.00024	0.0072	0.44	200.78
Potassium (K^+^)	1.3	0.00013	0.0038	0.61	291.71
Chloride (Cl^−^)	1.2	0.00011	0.0032	0.67	319.05
Bicarbonate (HCO_3_^−^)	2.2	0.00036	0.0107	0.36	157.97
Other cations (C^2+^)	3.1	0.00071	0.0209	0.25	102.44
Urea (U)	1.8	0.00024	0.0072	0.44	200.78
Creatinine (Cr)	2.6	0.00050	0.0148	0.30	128.46
Albumin (Alb)	35.5	0.08260	0.9442	0.012	0.0082
Globulins (Glob)	69.9^a^	0.27320	1.0000	0.003	0.0000

For ultrasmall pores σ_UP_ = 1 and PS_UP_ = 0 for all solutes.

^a^Average hydrodynamic radius of non-albumin plasma proteins calculated from data on individual globulin fractions and fibrinogen^28–30^.

To better describe the contribution of small ions to the osmotic pressure gradient across the capillary pores during HD, contrary to our previous work, in which we used the same osmotic coefficient for all ions (0.93), in this study we used separate osmotic coefficients as follows: Na 0.94, K 0.92, Cl 0.93, HCO_3_ 0.96, other cations (Ca^2+^, Mg^2+^) 0.80, other anions (SO4^2−^, H2PO4^−^, HPO4^2−^, PO4^3−^) 0.70^20^.

### Initial conditions

The model was defined for the steady-state conditions of a pre-dialysis virtual patient with several parameters modified with respect to a reference healthy man based on the literature data^17^ (see Table 2). In terms of fluid overload, knowing that dialysis patients accumulate the excess fluid almost exclusively in the extracellular fluid^2^, for simplicity we assumed that all excess fluid (2 L) goes to the interstitial fluid, and hence we simply increased the initial volume of the interstitial compartment with respect to the normal man, leaving all vascular compartments unchanged (thus assuming normal blood pressures in all compartments)^17^. The composition of the interstitial fluid (i.e. its water fraction and the concentrations of all solutes), the mean capillary blood pressure, and the Gibbs-Donnan coefficient for monovalent cations (α) were all found numerically to obtain initial steady-state conditions of the whole system based on the assumed composition of plasma (see Table 2) and the normal lymphatic absorption (8 L/day)^17^.

**Table 2:** Assumed patient characteristics^17^.

	Normal	pre-dialysis
**Plasma solutes**^**a**^		
Sodium (Na^+^) (mmol/L)	140	138^b^
Potassium (K^+^) (mmol/L)	4.3	5
Chloride (Cl^−^) (mmol/L)	103	103
Bicarbonate (HCO3^−^) (mmol/L)	26	22
Other cations (Ca^2+^, Mg^2+^) (mmol/L)	2	2
Urea (mmol/L)	6	27
Creatinine (mmol/L)	0.1	1
Albumin (g/dL)	4.1	3.75
Total protein (g/dL)	7.0	6.5
Plasma water fraction (–)	0.94	0.945
Hematocrit (%)	47	35
Cardiac output (L/min)	5.25	6.2
Fistula blood flow ( mL/min)	–	950

^a^For small ions, the concentrations listed above are assumed to be the concentrations of free ions that exert osmotic pressure, thus excluding the fractions of ions bounded to proteins or paired/complexed with other ions (those fractions are likely of similar order of magnitude on both sides of capillary walls).

^b^An average pre-dialysis plasma sodium level typically seen in hemodialysis patients^31,32^.

### Hemodialysis procedure

We analyzed a standard 4-h HD session including the pre-dialysis procedure of filling the extracorporeal circuit with the patient’s blood, with the priming fluid (normal saline) infused to the patient, and a couple of minutes of idle circulation before the start of the actual dialysis^17^. The assumed volume of the extracorporeal circuit was 220 mL and the dialyzer ultrafiltration was set to 9.25 mL/min to remove all excess fluid including the priming volume (2.22 L). The assumed composition of the dialysis fluid was as follows: Na^+^ 140 mmol/L, K^+^ 2 mmol/L, Cl^−^ 108 mmol/L, HCO3^−^ 34 mmol/L, other cations (Mg^2+^, Ca^2+^) 2 mmol/L. For all parameters of the HD procedure, including the clearances/dialysances of individual solutes, see our earlier work^17^.

### Sensitivity analysis

Given that this study focuses ultimately on the cardiovascular response to HD, the mean arterial pressure in the large arteries (MAP) at the end of a 4-h HD session was treated as the main model output for which the sensitivity to small changes in the values of individual model parameters was investigated. The dimensionless relative local sensitivity of the model output y (MAP) to each studied parameter, θ_k_, was calculated as follows^33,34^:

1 $$S_{k} = \left. {\frac{{\partial y}}{{\partial \theta _{k} }} \cdot \frac{{\theta _{k} }}{{y_{{}} }}} \right|_{{\theta _{k} = \theta _{{k,0}} }} ,\quad \theta _{k} ,y \ne 0$$

where the derivative was computed using the central difference approximation with the parameter θ_k_ increased and decreased by 0.01%.

The primary sensitivity analysis was restricted to the parameters related to the 3PM of the transcapillary fluid and solute transport (27 parameters in total): whole-body hydraulic conductivity of capillary walls (LpS), fractions of LpS contributed by the i-th type of pores (α_i_), solute hydrodynamic radii (r_s_), pore radii (r_i_), osmotic coefficients of small ions (φ_s_), molecular weight of proteins (MW_p_), density of proteins (ρ_p_), electric charge of albumin, globulins (non-albumin proteins) and “other anions”. Note that some of these parameters are used directly in the model equations, whereas some others, such as solute and pore radii, are used to calculate the equation parameters, such as solute reflection coefficients (σ_s_) and solute permeability-surface products (PS_s_)—see the Supplementary Information S1.

Additionally, we performed a separate sensitivity analysis with respect to all other model parameters (137 in total), including those describing the cardiovascular and extravascular compartments, baroreflex mechanisms, cardiac function, red blood cells, and tissue cells, as well as parameters related to the dialysis session, such as ultrafiltration rate, dialyzer blood flow rate, volume of the dialyzer and dialyzer tubing, composition of the dialysis fluid, and solute clearances/dialysances (the values of all these parameters and the model equations that use them may be found in our previous work^17^).

## Results

### Pre-dialysis steady state

The characteristics of the whole-body transcapillary water and protein transport across different types of pores under pre-dialysis steady-state conditions are shown in Table 3. We also compared the pre-dialysis steady-state conditions obtained for different fractions of total hydraulic capillary conductivity contributed by ultrasmall pores (α_UP_), keeping the large pore fraction (α_LP_) unchanged at 0.05 (see Table 4). We then performed a similar analysis of the impact of large pore fraction, keeping the ultrasmall pore fraction at 0.10 (see Table 5).

**Table 3:** Transcapillary water and protein transport (from blood to tissues) across large (LP), small (SP) and ultrasmall (UP) pores under pre-dialysis steady-state conditions.

	LP	SP	UP	Total
Fluid filtration, mL/h	178.2	196.3	6.1	380.6
Water filtration, mL/h	171.8	196.1	6.1	374.0
Albumin diffusion, mol/h	11.84	8.08	-	19.92
Globulins diffusion, mol/h	1.06	–	–	1.06
Albumin convection, mol/h	78.07	4.54	–	82.61
Globulins convection, mol/h	19.90	–	–	19.90
Albumin escape rate, %/h	4.69	0.66	–	5.35
Globulins escape rate, %/h	3.68	–	–	3.68
Mean capillary pressure, mmHg	15.43			
Interstitial fluid pressure, mmHg	0.12			
Interstitial-to-plasma albumin ratio	0.49			
Interstitial-to-plasma globulins ratio	0.34

**Table 4:** Transcapillary water and protein transport (from blood to tissues) under pre-dialysis steady-state conditions with different fractions of total hydraulic conductivity (LpS) contributed by ultrasmall pores (α_UP_) and the contribution of large pores (α_LP_) kept at 0.05.

	α_UP_					
	0.05	0.10	0.20	0.30	0.40	0.50
Filtration through LP, %	46.7	46.8	47.2	47.5	48.0	48.6
Filtration through SP, %	52.6	51.6	49.4	47.1	44.6	42.2
Filtration through UP, %	0.8	1.6	3.5	5.4	7.4	9.2
Albumin escape rate, %/h	5.37	5.35	5.33	5.31	5.29	5.28
Globulins escape rate, %/h	3.66	3.68	3.70	3.73	3.77	3.81
Mean capillary pressure, mmHg	15.38	15.43	15.52	15.63	15.76	15.92
Interstitial-to-plasma albumin ratio	0.50	0.49	0.49	0.49	0.49	0.49
Interstitial-to-plasma globulins ratio	0.34	0.34	0.34	0.34	0.35	0.35

**Table 5:** Transcapillary water and protein transport (from blood to tissues) under pre-dialysis steady-state conditions with different fractions of total hydraulic conductivity (LpS) contributed by large pores (α_LP_) and the contribution of ultrasmall pores (α_UP_) kept at 0.10.

	α_LP_					
	0.03	0.04	0.05	0.06	0.07	0.08
Filtration through LP, %	32.5	40.2	46.8	52.4	57.3	61.4
Filtration through SP, %	65.2	57.8	51.6	46.2	41.5	37.6
Filtration through UP, %	2.2	1.9	1.6	1.4	1.2	1.0
Albumin escape rate, %/h	4.06	4.76	5.35	5.86	6.30	6.69
Globulins escape rate, %/h	2.55	3.16	3.68	4.12	4.50	4.82
Mean capillary pressure, mmHg	17.80	16.55	15.43	14.43	13.53	12.73
Interstitial-to-plasma albumin ratio	0.37	0.44	0.49	0.54	0.58	0.62
Interstitial-to-plasma globulins ratio	0.24	0.29	0.34	0.38	0.41	0.45

### Hemodialysis

**Figure 3:**
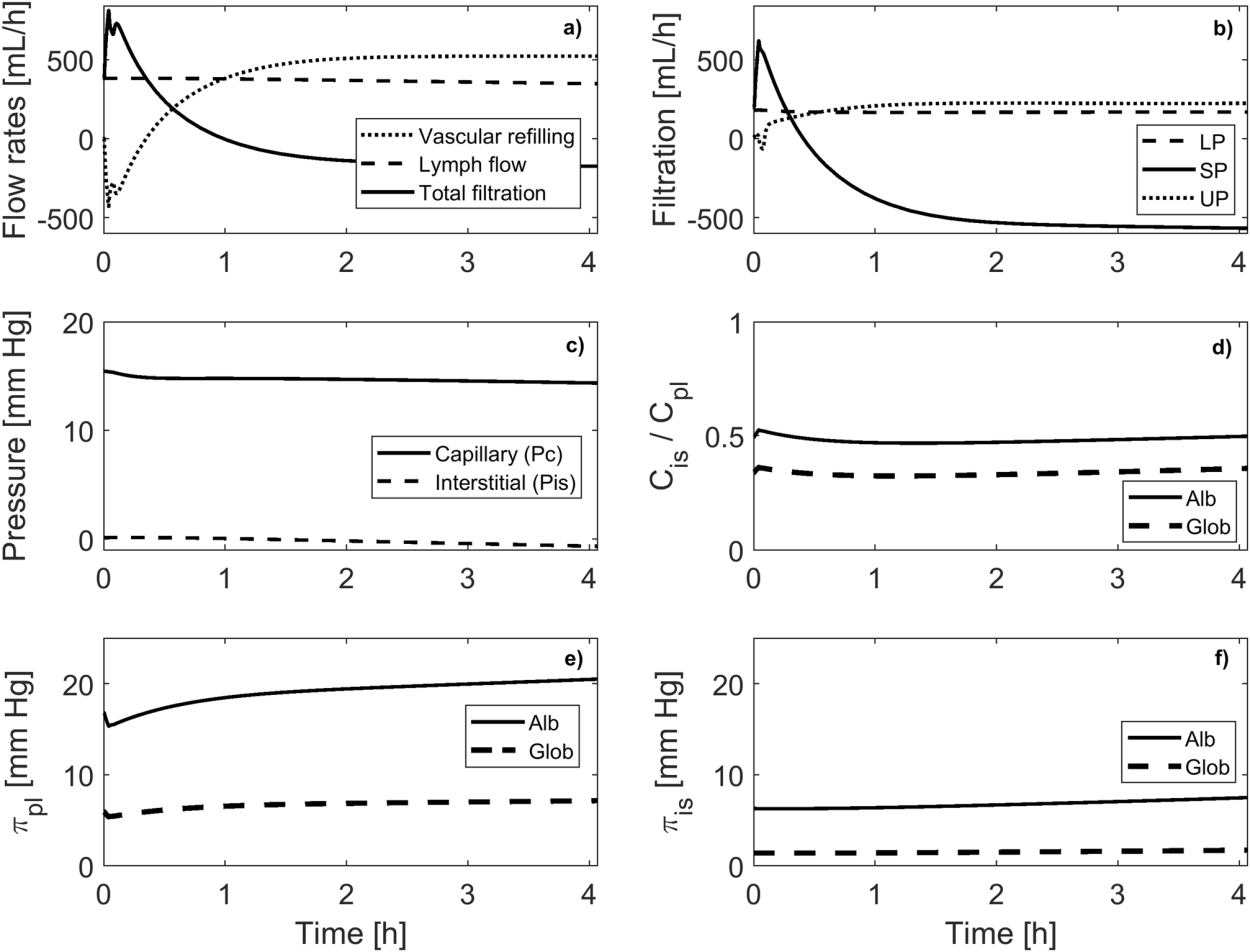
Model-simulated changes in transcapillary transport variables during hemodialysis. (**a**) Vascular refilling as a difference between lymphatic absorption and total transcapillary filtration; (**b**) transcapillary fluid filtration flows from blood to tissues through large (LP), small (SP), and ultrasmall (UP) pores; (**c**) mean capillary blood pressure (P_sc_) and interstitial hydrostatic pressure (P_is_); (**d**) interstitial-to-plasma concentration ratio for albumin and globulins; (**e**) plasma oncotic pressure exerted by albumin and globulins; (**f**) interstitial oncotic pressure exerted by albumin and globulins.

During the simulated HD procedure the transcapillary fluid filtration is subject to substantial changes (see Fig. 3a). At the beginning of the procedure, when the priming saline is infused to the virtual patient, the total filtration increases, which is due to the relatively sudden reduction in the plasma oncotic pressure exerted by albumin and globulins following hemodilution by the saline (see Fig. 3e). During HD the total transcapillary filtration from blood to tissues progressively decreases until it eventually reverses into transcapillary absorption of fluid from the interstitial space at circa 1 h into the dialysis session. This results mainly from the reversion of flow direction through the small pores due to increasing plasma oncotic pressure induced by dialyzer ultrafiltration combined with a decrease in capillary blood pressure—see Fig. 3c,e (note that the reversion of flow direction through small pores occurs circa 0.5 h earlier than that of the total transcapillary filtration). The fluid filtration across the large pores decreases to a much lower extent given that the oncotic pressure gradient across the large pores is much lower due to low reflection coefficients of albumin and globulins (see Table 1). The changes in water filtration across ultrasmall pores are opposite to those for small pores (a progressive increase), which is due to the fact that the reduced leakage of negatively charged proteins during HD entails the increased transcapillary flow of “other anions”, which in the model are assumed to move according to the electroneutrality condition—this leads to the extra osmotic pressure gradient developing across ultrasmall pores, which draws more vascular water through this transport channel.

As fluid is removed from the body via ultrafiltration, the volume and the pressure of the interstitial fluid decreases (see Fig. 3c). This leads to a progressive increase in the interstitial oncotic pressure (see Fig. 3f), which partly reduces the forces driving transcapillary fluid absorption (the vascular refilling mechanism). It also leads to a slight reduction in the lymph absorption. The interstitial-to-plasma ratio of albumin remains during HD relatively stable at below 0.5 (see Fig. 3d). The analogous ratio for globulins is slightly below 0.4. Both values are within the range reported in the literature^4,35–37^).

**Figure 4:**
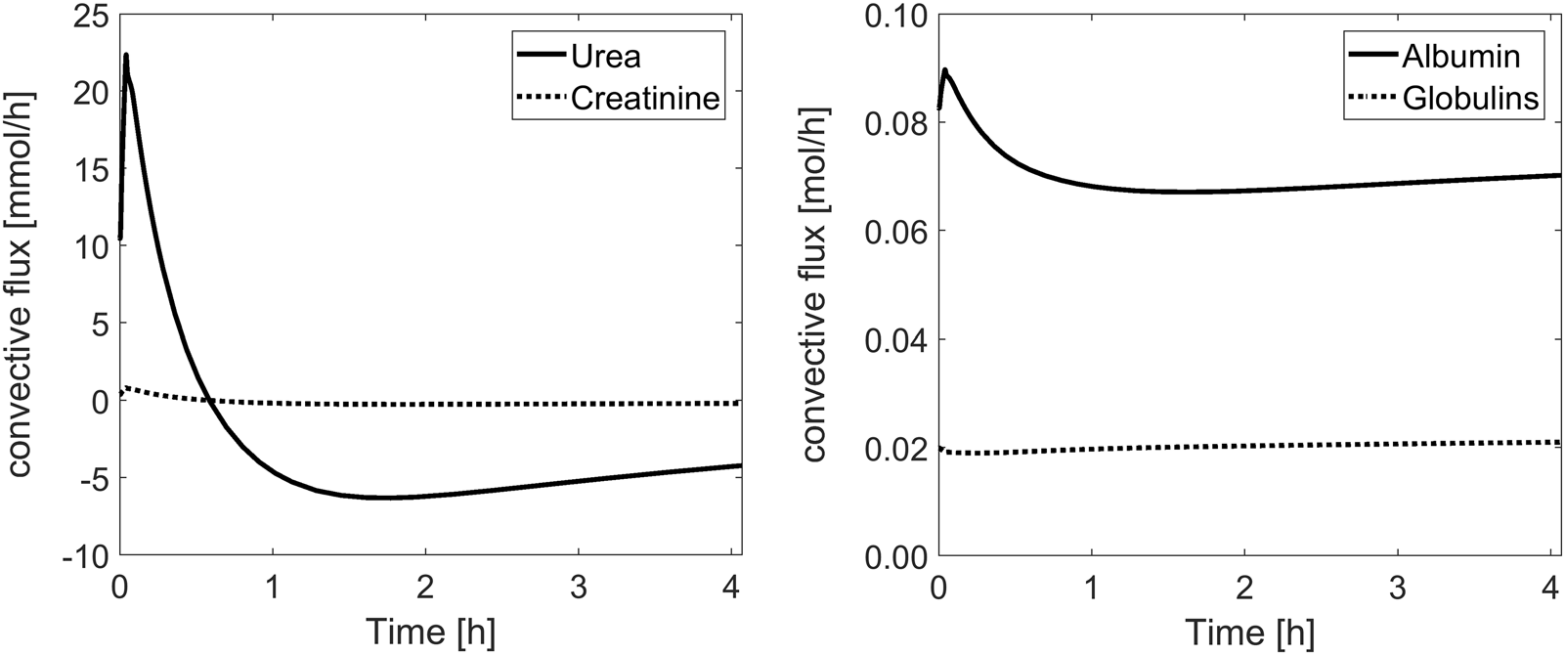
Model-simulated transcapillary convective fluxes of albumin, globulins, urea and creatinine during hemodialysis procedure.

Given the substantial changes in the magnitude and direction of transcapillary water flows through small pores (see Fig. 3b), the convective fluxes of individual solutes are also subject to intradialytic changes, as shown in Fig. 4. For small solutes and albumin, following the initial rise in the transcapillary convective flux (due to increased capillary filtration through small pores after the infusion of the priming saline), the transcapillary convection decreases due to decreasing filtration through small pores. For small solutes, after certain time (slightly over 0.5 h), the transcapillary convective leakage of solutes reverses into transcapillary absorption and remains so until the end of dialysis. For globulins, no initial rise in the convective flux can be observed, as these proteins travel only via large pores, for which the rate of filtration is not affected by the infusion of the priming saline.

**Figure 5:**
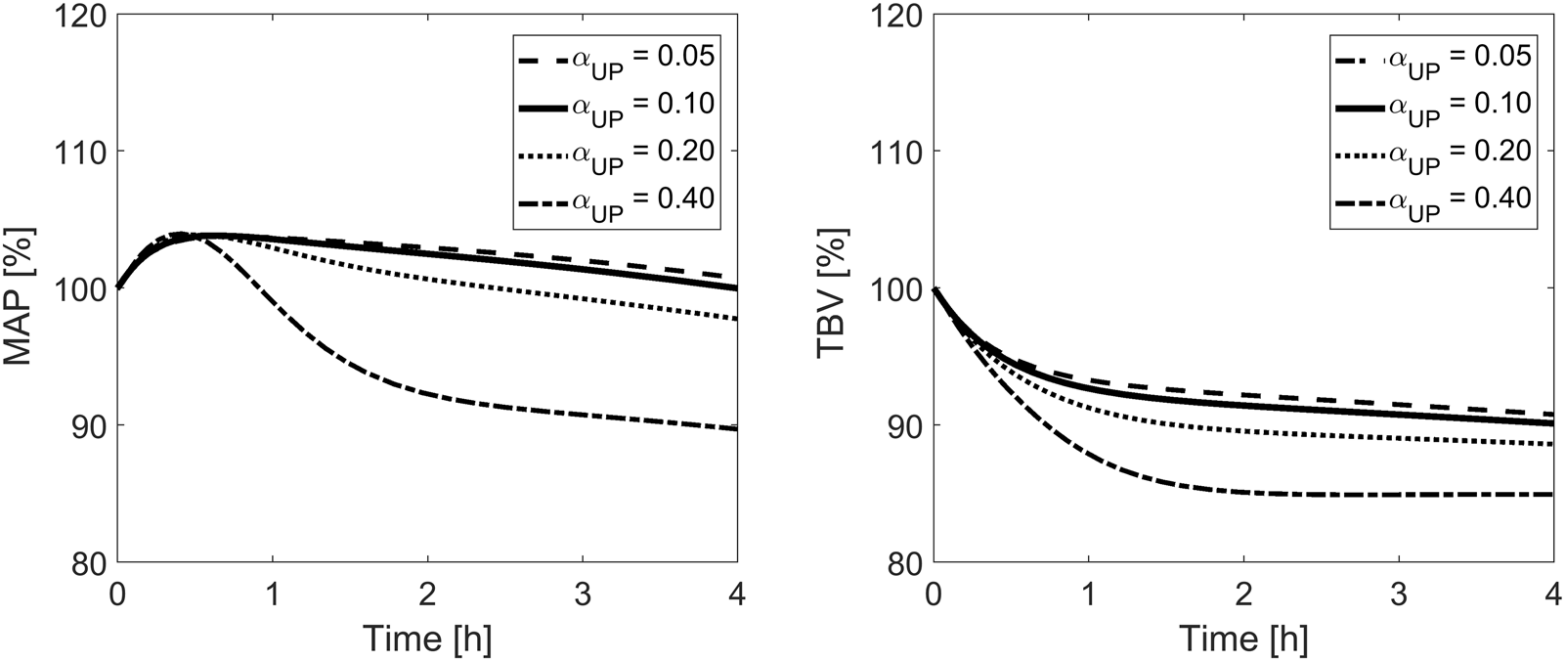
Model-simulated relative changes of mean arterial blood pressure (MAP) and total blood volume (TBV) during hemodialysis for different fractions of the capillary hydraulic conductivity attributed to ultrasmall pores (α_UP_) with the fraction attributed to large pores kept unchanged at the basal value of 0.05.

**Figure 6:**
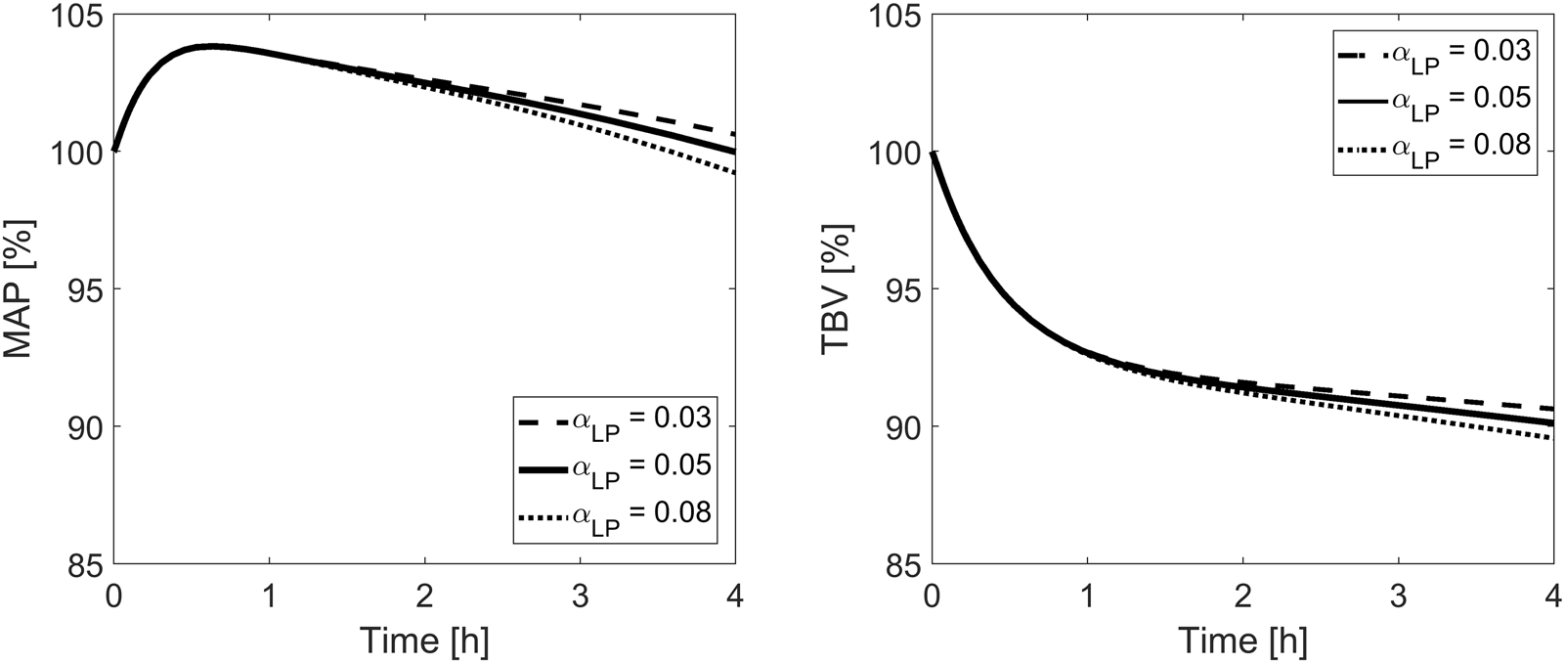
Model-simulated relative changes of mean arterial blood pressure (MAP) and total blood volume (TBV) during hemodialysis for different fractions of the capillary hydraulic conductivity attributed to large pores (α_LP_) with the fraction attributed to ultrasmall pores kept unchanged at the basal value of 0.10.

The relative changes in mean arterial blood pressure (MAP) and total blood volume (TBV) during the simulated HD (without the pre-dialysis procedure of filling the extracorporeal circuit) for different fractions of total capillary hydraulic conductivity attributed to ultrasmall pores (α_UP_) or large pores (α_LP_) are shown in Figs. 5 and 6.

### Sensitivity analysis

**Figure 7:**
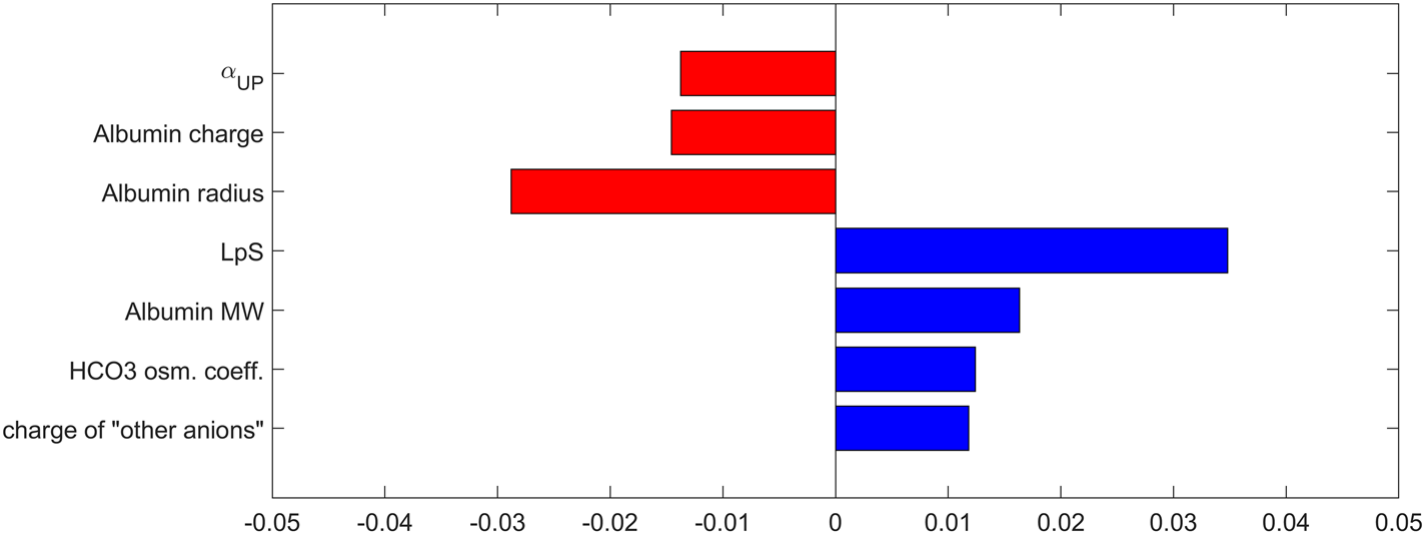
Relative sensitivity of the end-of-dialysis mean arterial pressure (MAP) to model parameters related to the three-pore model of the capillary wall. Only parameters with the relative sensitivity greater than 0.01 are shown.

In Fig. 7, we show the results of our sensitivity analysis, i.e., the relative sensitivity of the mean arterial pressure at the end of the simulated 4-h HD session with respect to model parameters related to the 3PM of the capillary wall. For clarity, we only show the parameters with the associated relative sensitivity greater than 0.01. For all 27 studied parameters the relative sensitivities are lower than 0.04 (in absolute terms) meaning that a 10% change in the parameter value would lead to less than 0.4% change in the studied model output (MAP). Among the studied parameters, the relatively highest impact on the end-of-dialysis MAP was found for the whole-body hydraulic conductivity of capillary walls (LpS), fraction of LpS contributed by ultrasmall pores (α_UP_), parameters related to albumin (radius, charge and MW), osmotic coefficient of bicarbonate ions, and charge of “other anions” (assumed −2) whose transport across the capillary wall in the model is governed by electroneutrality condition and hence is related to the transport of negatively charged proteins (mainly albumin).

**Figure 8:**
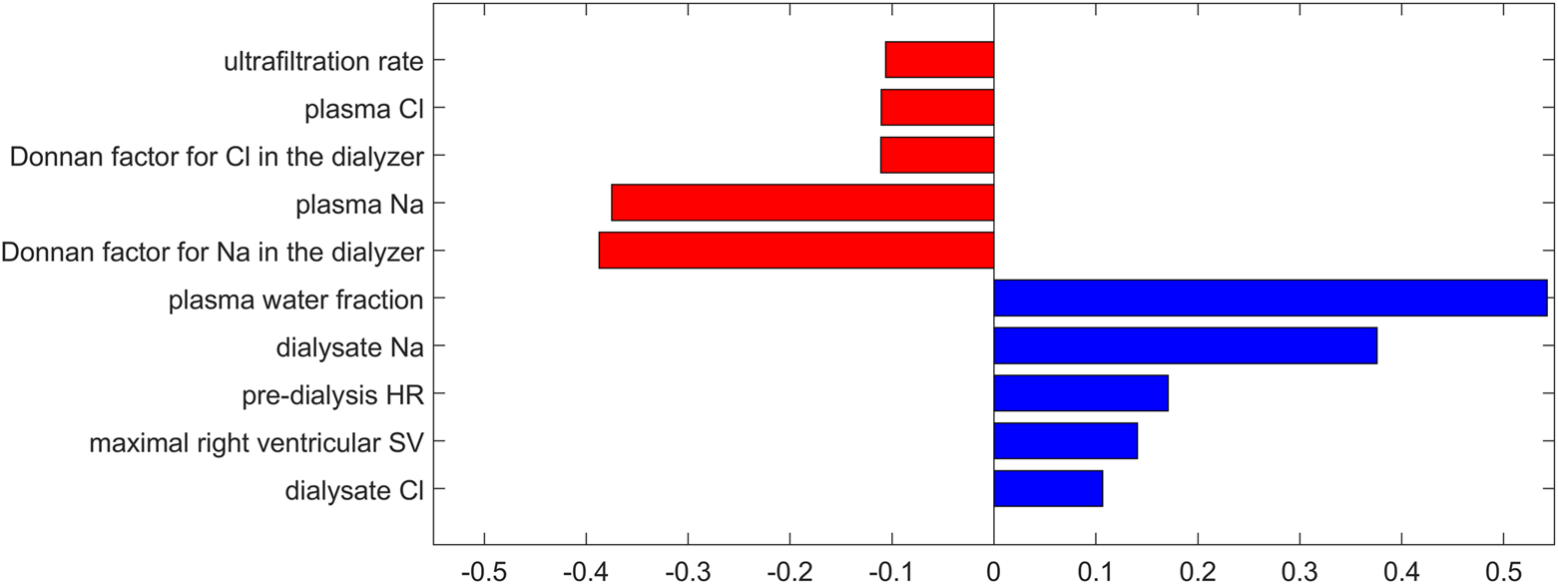
Relative sensitivity of the end-of-dialysis mean arterial pressure (MAP) to model parameters other than those related to the three-pore model of the capillary wall. Only parameters with the relative sensitivity greater than 0.1 are shown.

Figure 8 shows the analogous relative sensitivities of the end-of-dialysis mean arterial pressure to 137 other model parameters or dialysis settings, as mentioned in the Methods section. For clarity, we only show the parameters with the associated relative sensitivity greater than 0.1 (among the parameters not shown, 99 parameters were associated with relative sensitivities lower than 0.01). The relatively highest impact on the end-of-dialysis MAP was found—unsurprisingly—for the ultrafiltration rate and the parameters related to the transport of sodium and chloride across the dialyzer membrane (concentrations in plasma and dialysis fluid, plasma water fraction, and the Donnan factors), and—somewhat less intuitively—for a couple of parameters related to the cardiac function (initial heart rate and the maximal stroke volume of the right ventricle).

## Discussion

As far as we know, the model presented in this study is the first compartmental model of the human cardiovascular system with baroreflex regulation integrated with the model of whole-body water and solute transport that takes into account the structural (porous) properties of the capillary endothelium.

Due to substantial dialysis-induced changes in the transcapillary flow conditions through individual types of capillary pores, particularly through small and ultrasmall pores, the convective transport of solutes across the capillary walls is also subject to intradialytic changes. This is particularly important for the transcapillary transport of proteins known to occur mostly by convection^18^. As the magnitude and direction of flows through different types of capillary pores change during HD, the effective reflection or sieving coefficients for individual solutes also change, and hence the classic homogeneous membrane model, which assumes constant reflection coefficients, would not be able to accurately describe the aforementioned changes in the convective transport of proteins, particularly in the case of albumin, which can pass through both large and small pores.

As far as the 3PM is concerned, the most challenging aspect of using this model is to establish the relative number of pores of different size and to divide among them the assumed total capillary hydraulic conductivity (LpS). This is even more challenging in the studies such as ours, where we consider transcapillary fluid and solute exchange on the whole-body level with a lumped (average) capillary wall representing capillary walls from all body tissues. It is particularly difficult to assign the hydraulic capacity of ultrasmall pores that are present only in the endothelium of continuous capillaries (assumed in this study for the whole body), thus not existing in other types of capillaries (less common on the whole-body level). Based on data by Rippe and Haraldsson^18^ and Michel and Curry^38^, in the base scenario we attributed 5% of LpS to large pores and 10% to ultrasmall pores, with the remaining 85% attributed to small pores. However, much higher values for the UP fraction in various tissues were proposed by other authors, in particular ~ 30% estimated by Kellen and Bassingthwaighte^39^, 40% claimed by Wolf^13^ or 50% suggested by Pappenheimer^40^. According to our study, the fraction of LpS contributed by ultrasmall pores on the whole-body level (α_UP_) has very little influence on the transcapillary steady-state conditions. Changing α_UP_ between 0.05 and 0.50 led to marginal changes in the observed transcapillary escape rate of albumin (TER_Alb_) or mean capillary blood pressure, despite an increase in water filtration through UP from 0.8 to 9.2% of the total filtration (see Table 4). On the other hand, during HD, which induces extensive systemic perturbations in terms of fluid and solute imbalances, α_UP_ plays a much more important role (see Fig. 5). From the results of our study, it seems that high values of α_UP_ (0.4 or higher) are rather unlikely in an otherwise healthy dialysis patient, as they lead to a significantly larger decrease in the simulated values of TBV and MAP—much greater than should be expected for the assumed (mostly normal) parameters of the cardiovascular system and the relatively moderate ultrafiltration of 2 L. This can be also interpreted as an indication that patients with a high value of α_UP_, i.e. a relatively large number of ultrasmall pores on the whole-body level (e.g. due to large amount of muscle or fat tissue), may be possibly more prone to intradialytic hypotension. This hypothesis could be further investigated in the future by attempting to assess the relative amounts of tissues with continuous capillaries versus tissues with other types of capillaries in patients undergoing HD therapy. Note that the importance of ultrasmall pores in our analysis of HD results mainly from the osmotic pressure gradient exerted by “other anions”, which during HD travel in the model in the opposite direction to proteins in order to keep capillary plasma and interstitial fluid electroneutral. This may be an oversimplification, as there may be more complex processes and mechanisms affecting the distribution of electrical charge across the capillary wall following a reduced leakage of negatively charged proteins. Nevertheless, several studies have shown the important role of ultrasmall pores (or aquaporins) in non-steady conditions, for instance during peritoneal dialysis^14,41–44^.

The results are somewhat opposite for the fraction of large pores (α_LP_). Changing α_LP_ between 0.03 and 0.08 led to relatively minor changes in the hemodynamic response to HD (see Fig. 6) but provided largely different initial steady states of the modeled system with filtration through large pores changing between 32 and 61% of the total filtration (see Table 5). Looking at the steady-state values of TER_Alb_ and the mean capillary pressure obtained for different α_LP_, it seems that the value chosen for our base scenario (α_LP_ = 0.05) is quite likely with TER_Alb_ ≈ 5%/h, P_sc_ ≈ 15.5 mmHg and interstitial-to-plasma albumin ratio below 0.5. A similar value for α_LP_ was obtained in the previous study from our group^37^ (although that study assumed α_SP_ = 0.60).

The importance of the parameter α_UP_ was confirmed in our sensitivity analysis, where it was found to be one of the parameters of the 3PM with the relatively highest influence on the end-of dialysis MAP. It should be noted that, as shown in Figs. 7 and 8, several other parameters of the model have a relatively higher impact on the end-of-dialysis MAP compared to α_UP_; however, the values of those parameters are unlikely to differ largely from the values assumed in the model (perhaps with the exception for LpS), and hence, even if the model seems to be more sensitive to those parameters, their relatively low physiological range reduces their relative importance in the model. The value of α_UP_, on the other hand, carries a much higher uncertainty, as shown by the largely different values provided in the aforementioned studies (e.g. 50% vs 10%), which justifies our model-based investigation of how the value of this parameter affects the cardiovascular response to HD. Note also that the generally low values of calculated relative sensitivities of MAP to all model parameters indicate that our model is relatively robust and resistant to isolated changes of individual parameter values.

It should be noted that patients on hemodialysis often have various cardiovascular diseases or conditions, such as hypertension or autonomic dysfunction, and hence the pre-dialysis pressures and volumes of individual cardiovascular compartments as well as the parameters of the baroreflex mechanisms (gains, amplitudes, and time constants) may be different than those assumed in our model for a healthy man. In consequence, the cardiovascular response to HD in such patients, in particular intradialytic changes of the mean arterial blood pressure, may differ from that presented by our simulations. Our sensitivity analysis showed that four baroreflex parameters (out of 17) have a similar or higher impact on the end-of-dialysis MAP compared to α_UP_ (amplitude of the peripheral resistance regulation and gains of the mechanisms controlling peripheral resistance, venous unstressed volume and heart contractility). Therefore, substantial changes in the values of these baroreflex parameters (e.g. a substantial reduction of the gain or amplitude of peripheral resistance regulation) may have a similar effect on dialysis-induced changes in MAP as that suggested for a high fraction of ultrasmall pores.

In this study, we assumed a pre-dialysis plasma sodium level of 138 mmol/L and a common dialysate sodium concentration of 140 mmol/L^31,32^. With these values, taking into account the corrections for plasma water fraction and the Gibbs–Donnan effect, there will be a slight diffusion of sodium from the dialysis fluid to the patient. With the use of a higher dialysate sodium (e.g. in patients with hyponatremia or intradialytic hypotensive symptoms, such as muscle cramps), the intradialytic sodium diffusion would be accordingly higher, which, as shown by the sensitivity analysis, would obviously impact the intradialytic changes of blood pressure. However, given the relatively high rates of transcapillary diffusion of small solutes, such as sodium, and relatively small intradialytic changes in plasma sodium (compared, for instance, to changes in plasma urea concentration), this would not affect our conclusions with regard to the relative impact of the fraction of ultrasmall pores.

Our study has, however, certain limitations. Firstly, as already mentioned, we analyze the transcapillary transport of water and solutes on the whole-body level assuming that the properties of the capillary walls are the same across the entire body (including pulmonary capillaries) and equivalent to those of continuous capillaries found in skeletal muscles, thus we do not take into account the fact that different organs and tissues may have different microvascular properties and hence different rates of local transcapillary fluid and solute exchange during HD. Similarly, in our study, the lymph absorption from the interstitial space reflects the whole-body lymph flow, including the pulmonary lymph. However, the properties of the pulmonary capillary walls and the pulmonary interstitial fluid differ from those of other tissues^45^. Secondly, we assume that all parameters describing the transcapillary transport processes are the same for both sides of the capillary wall (e.g. the same solute reflection coefficients regardless of flow direction) and that they are constant. During HD, however, the properties of the capillary walls may potentially change, thus influencing the analyzed transport processes. Moreover, we ignore the recent theory on the protein gradient developing between the bulk interstitial fluid and the fluid beneath the so-called glycocalyx^3,46^, according to which, when calculating the Starling forces across the capillary wall, one should use the (lower) oncotic pressure of the subglycocalyx fluid and not that of the bulk interstitial fluid^2,3^. Finally, our model of baroreflex regulation is also subject to some limitations which we discuss in our earlier work^17^.

In summary, our study showed that during non-steady conditions, such as HD, the 3PM reveals complex transcapillary transport phenomena, which are not possible to be captured by a standard homogeneous membrane model with fixed parameter values. We also showed that the fraction of hydraulic conductivity attributed to ultrasmall pores may play an important role in the transcapillary water transport during HD and the cardiovascular response to HD. It seems hence, that the 3PM—with a particular attention to the ultrasmall pore fraction—should be the preferred conceptual model for detailed investigations of vascular refilling mechanisms, the rate of capillary protein leakage, and the maintenance of cardiovascular stability during HD.

## Supplementary information

Supplementary Information

## Data Availability

The detailed description of the mathematical model used in this study as well as the values of all model parameters and initial conditions may be found in our previous work, as mentioned in the manuscript.
