# Supplementary material for: Transcapillary transport of water, small solutes and proteins during hemodialysis
Source: Sci Rep. 2020 Oct 30;10:18736. doi: 10.1038/s41598-020-75687-1 (PMC7603324; doi:10.1038/s41598-020-75687-1)
Supplement: Supplementary file 1 — Supplementary Information [file 41598_2020_75687_MOESM1_ESM.pdf]

Leszek Pstras, Jacek Waniewski, Bengt Lindholm
"Transcapillary transport of water, small solutes and proteins during hemodialysis"

SUPPLEMENTARY MATERIAL

THREE-PORE MODEL OF THE CAPILLARY WALL

Fluid filtration from the capillary compartment to the interstitial compartment depends on the imbalance between the Starling forces acting across the capillary walls [1]: the hydraulic capillary blood pressure, the hydrostatic interstitial pressure and the osmotic (mainly oncotic) pressures exerted by all solutes on both sides of the capillary wall [2,3]. In the three-pore model (3PM) the transcapillary filtration needs to be defined separately for each type of pore (J_i) as follows:

$$J_i = \alpha_i LpS\left[\left(P_{sc} - P_{is}\right) - \sum_p \sigma_{p,i}\left(\pi_{p,pl,sc} - \pi_{p,is}\right) - \sum_s \sigma_{s,i}\varphi_s\left(\alpha_s \frac{c_{s,pl,sc}}{F_{pl,sc}} - \frac{c_{s,is}}{F_{is}}\right)RT\right] \quad (1)$$

where LpS is the whole-body hydraulic conductivity of capillary walls (assumed constant), α_i is the fraction of LpS contributed by the i-th type of pore ($\alpha_{LP} + \alpha_{SP} + \alpha_{UP} = 1$), P_{sc} is the mean hydraulic pressure of systemic capillary blood, P_{is} is the hydrostatic pressure of the interstitial fluid, $\sigma_{p,i}$ and $\sigma_{s,i}$ are the Staverman's reflection coefficients of protein p and solute s at the i-th pore, $\pi_{p,pl,sc}$ and $\pi_{p,is}$ are the oncotic pressures (colloid osmotic pressures) of protein p (albumin or globulins) in the capillary plasma and interstitial fluid, φ_s is the osmotic activity coefficient of solute s, α_s is the Gibbs-Donnan coefficient for ion s with charge z_s (for simplicity $\alpha_s=\alpha^{Zs}$ [8], where α is determined from the steady-state conditions), and RT is a constant (= 19.3 mmHg/mmol/L).

The total transcapillary fluid filtration is then expressed as:

$$J = J_{LP} + J_{SP} + J_{UP} \quad (2)$$

It was assumed that the mean hydraulic pressure of capillary plasma (P_{sc}) is resistant to isolated changes in arterial pressure (the auto-regulatory capacity of the capillary bed), whereas 80% of changes in venous pressure are transmitted to the capillaries [4]. P_{sc} is hence calculated as:

$$P_{sc} = P_{sc,0} + w_v \cdot (P_{sv} - P_{sv,0}) \quad (3)$$

where $P_{sc,0}$ is the initial mean capillary pressure calculated from the initial steady-state conditions, w_v is a parameter (assumed value of 0.8, based on experimental data [4]) and $P_{sv,0}$ is the assumed initial (normal) pressure in the small veins compartment (12 mm Hg [4]).

The hydrostatic pressure of the interstitial fluid was described as a linear function of the interstitial volume [2,5]:

$$P_{is} = P_{is,n} + \frac{1}{C_{is}} \cdot \left(V_{is} - V_{is,n}\right) \quad (4)$$

where $P_{is,n}$ is the normal interstitial pressure corresponding to the normal interstitial volume ($V_{is,n}$) and C_{is} is the interstitial compliance, which was assumed to be 12% of normal interstitial volume per mm Hg [6].

The plasma oncotic pressure (in mm Hg) exerted by albumin ($\pi_{alb,pl,sc}$) and globulins ($\pi_{glob,pl,sc}$) are calculated using the following equations [4] (based on Landis-Pappenheimer equations [7]).

$$\pi_{alb,pl,sc} = a_{sc}\left(2.8c_{p,sc} + 0.18{c_{p,sc}}^{2} + 0.012{c_{p,sc}}^{3}\right) \tag{5}$$

$$\pi_{glob,pl,sc} = b_{sc}\left(1.1c_{p,sc} + 0.13{c_{p,sc}}^{2} + 0.005{c_{p,sc}}^{3}\right) \tag{6}$$

where $c_{p,sc}$ is the total protein concentration in capillary plasma in g/dL, whereas a_{sc} and b_{sc} are variable albumin and globulins mass fractions of total plasma proteins ($a_{sc} + b_{sc} = 1$).

Similar equations were used for calculating the oncotic pressure of interstitial albumin and globulins based on the total concentration of proteins in the interstitial compartment ($c_{p,is}$) and the corresponding a_{is} and b_{is} fractions (also variable).

The transcapillary transport of small solutes (except other anions) through the i-th type of pore can be described as a sum of diffusive and convective flows using the following equation [4]:

$$Q_{s,i} = PS_{s,i}\left(\alpha_s \frac{c_{s,pl,sc}}{F_{pl,sc}} - \frac{c_{s,is}}{F_{is}}\right) + J_{w,i}S_{s,i}\left[\left(1-f_{s,i}\right)\alpha_s \frac{c_{s,pl,sc}}{F_{pl,sc}} + f_{s,i}\frac{c_{s,is}}{F_{is}}\right] \tag{7}$$

where $PS_{s,i}$ is the permeability-surface product for solute s at the i-th type of pore (assumed constant), α_s is the Gibbs-Donnan coefficient for ion s, $c_{s,pl,sc}$ and $c_{s,is}$ are the concentrations of solute s in the capillary plasma and interstitial fluid, $F_{pl,sc}$ and F_{is} are variable water fractions of the capillary plasma and interstitial fluid, $J_{w,i}$ is the water flow through i-th pore from capillaries to interstitium (calculated as the difference between fluid filtration and the volumetric flow of convective protein leakage), $S_{s,i}$ is the sieving coefficient for solute s at the i-th type of pore and $f_{s,i}$ is defined as follows [8]:

$$f_{s,i} = \frac{1}{Pe_{s,i}} - \frac{1}{\exp\left(Pe_{s,i}\right) - 1} \tag{8}$$

where $Pe_{s,i}$ is the modified Peclet number describing the relationship between the convective and diffusive transport of solute s through i-th type of pore [8] (if diffusion and convection are of opposite directions, the below equation takes the negative sign):

$$Pe_{s,i} = \frac{J_{w,i}S_{s,i}}{PS_{s,i}} \tag{9}$$

The sieving coefficient for each solute at the i-th type of pore is calculated as $S_{s,i} = 1-\sigma_{s,i}$, where [9]:

$$\sigma_{s,i} = \begin{cases} 1 - \dfrac{(1-\lambda_{s,i})^2 \cdot [2-(1-\lambda_{s,i})^2] \cdot (1-\frac{\lambda_{s,i}}{3})}{1 - \frac{\lambda_{s,i}}{3} + \frac{2}{3}{\lambda_{s,i}}^{2}} & \text{for } \lambda_{s,i} < 1 \\ 0 & \text{for } \lambda_{s,i} \geq 1 \end{cases} \tag{10}$$

where $\lambda_{s,i}$ is the ratio of solute and pore radii ($\lambda_{s,i} = r_s/r_i$).

Analogically, the transport of protein p through each type of pore is using the diffusive-convective equation [8,10]:

$$Q_{p,i} = PS_{p,i}\left(c_{p,pl,sc} - c_{p,is}\right) + J_i S_{p,i}\left[\left(1-f_{p,i}\right)c_{p,pl,sc} + f_{p,i}c_{p,is}\right] \quad (11)$$

where p denotes albumin or globulins, $PS_{p,i}$ is the permeability-surface product of the lumped capillary wall for protein p (assumed constant), J_i is the rate of fluid filtration from the capillaries to the interstitium through i-th type of pore, $S_{p,i}$ is the capillary sieving coefficient of protein p, and f_p is defined as in the equation (8).

The transport of other anions (A^{2-}) is calculated to obtain a zero net flow of charge across the capillary wall.

The volumetric flow of proteins from capillary plasma to interstitium through i-th type of pore is calculated as:

$$Q_{p,i,V} = Q_{p,i} \cdot \frac{MW_p}{\rho_p} \quad (12)$$

where MW_p is the molecular weight of protein p (assumed 69,000 g/mol for albumin and 170,000 g/mol for globulins [11,12]) and ρ_p is the protein density (assumed 1.37 g/cm^3 for both albumin and globulins [13]).

The permeability-surface product for solute s and the i-th type of pore is calculated from the following equation [9]:

$$PS_{s,i} = D_s\left(\frac{A_{0,i}}{\Delta x_i}\right)\left(\frac{A_i}{A_{0,i}}\right)_s \quad (13)$$

where D_s is the free diffusion coefficient of solute s calculated as [9]:

$$D_s = \frac{RT}{6\pi \cdot N_A \cdot r_S \cdot \eta_{H_2O}} \quad (14)$$

where N_A is the Avogadro number, r_s is the solute radius and η_{H2O} is water dynamic viscosity at 37 ^oC (=0.0007 Pa·s),

$A_{0,i}$ is the total cross-sectional area of pores of type i, A_i is the effective pore area available for restricted diffusion, $\left(\frac{A_{0,i}}{\Delta x_i}\right)$ is the total unrestricted pore area over unit diffusion length calculated as [9]:

$$\left(\frac{A_{0,i}}{\Delta x_i}\right) = \frac{8 \cdot \eta_{H_2O} \cdot Lp \cdot \alpha_i}{r_i^2} \quad (15)$$

and $\left(\frac{A_i}{A_{0,i}}\right)_s$ - the fraction of total pore area available for restricted diffusion - is calculated as [9]:

$$\left(\frac{A_i}{A_{0,i}}\right)_s = \frac{(1-\lambda_{s,i})^{\frac{9}{2}}}{1 - 0.3956 \cdot \lambda_{s,i} + 1.0616 \cdot \lambda_{s,i}{}^2} \quad (16)$$

REFERENCES

1. Guyton, A. C. & Hall, J. E., *Textbook of medical physiology*, 11th ed. (Elsevier Saunders, Philadelphia, PA, 2006).
2. Gyenge, C. C., Bowen, B. D., Reed, R. K. & Bert, J. L., Transport of fluid and solutes in the body I. Formulation of a mathematical model. *Am J Physiol Heart Circ Physiol* **277** (3 Pt 2), H1215-27 (1999).
3. Wolf, M. B., Whole body acid-base and fluid-electrolyte balance: a mathematical model. *Am J Physiol Renal Physiol* **305** (8), F1118-31 (2013).
4. Pstras, L. & Waniewski, J., *Mathematical modelling of haemodialysis: Cardiovascular response, body fluid shifts, and solute kinetics* (Springer Nature Switzerland AG, Cham, Switzerland, 2019).
5. Ursino, M. & Innocenti, M., Modeling arterial hypotension during hemodialysis. *Art Org* **21** (8), 873-890 (1997).
6. Aukland, K. & Reed, R. K., Interstitial-lymphatic mechanisms in the control of extracellular fluid volume. *Physiol Rev* **73** (1), 1-78 (1993).
7. Landis, E. M. & Pappenheimer, J. R., in *Handbook of physiology. Section 2: Circulation*, edited by Hamilton, W. F. & Dow, P. (American Pysiological Society, Washington, DC, 1963), Vol. 11.
8. Waniewski, J., Mathematical modeling of fluid and solute transport in hemodialysis and peritoneal dialysis. *J Membrane Sci* **274**, 24-37 (2006).
9. Rippe, B. & Haraldsson, B., Transport of macromolecules across microvascular walls: the two-pore theory. *Physiol Rev* **74** (1), 163–219 (1994).
10. Waniewski, J., in *Theoretical foundations for modelling of membrane transport in medicine and biomedical engineering* (Institute of Computer Science, Polish Academy of Sciences, Warsaw, 2015), pp. 24-7.
11. Feher, J., *Quantitative Human Physiology. An Introduction.* (Academic Press, Elsevier, Amsterdam, 2012).
12. Scatchard, G., Batchelder, A. C. & Brown, A., Chemical, clinical and immunological studies on the products of human plasma fractionation. VI. The osmotic pressure of plasma and of serum albumin. *J Clin Invest* **23** (4), 458-464 (1944).
13. Fischer, H., Polikarpov, I. & Craievich, A. F., Average protein density is a molecular-weight-dependent function. *Protein Sci* **13** (10), 2825–2828 (2004).
14. Rippe, B., Venturoli, D., Simonsen, O. & de Arteaga, J., Fluid and electrolyte transport across the peritoneal membrane during CAPD according to the three-pore model. *Perit Dial Int* **24** (1), 10-27 (2004).
